# Cannabis Synthetic Seeds: An Alternative Approach for Commercial Scale of Clonal Propagation and Germplasm Conservation

**DOI:** 10.3390/plants11233186

**Published:** 2022-11-22

**Authors:** Adel Zarei, Biruk A. Feyissa, Benjamin Davis, Elham Tavakouli Dinani

**Affiliations:** Safari Flower Co., 2818 House Road, Stevensville, ON L0S 1S0, Canada

**Keywords:** *Cannabis sativa* L., acetylsalicylic acid, in vitro propagation, micropropagation, synseeds

## Abstract

Indoor cannabis (*Cannabis sativa*) cultivation has been rapidly increasing in many countries after legalization. Besides conventional propagation through cuttings, synthetic seed production provides a competent system for mass propagation, germplasm conservation and international exchange of genetic materials. The present study developed a reliable protocol for cannabis synthetic seed production using encapsulation of nodal segments derived from in vitro or in vivo sources. Synthetic seeds were produced in 3% sodium alginate and 75 mM calcium chloride in Murashige and Skoog (MS) medium and stored under various environmental conditions for up to 150 days. The plantlets regrowth efficiency was monitored on culture media up to 30 days after the storage period. Regrowth rates of 70% and 90% were observed in synthetic seeds from in vitro and in vivo-derived sources, respectively, when stored in 6 °C under 50 μmol s^−1^ m^−2^ light for 150 days. Furthermore, addition of acetylsalicylic acid (ASA) to the encapsulation matrix not only postponed precocious germination of synthetic seeds at 22 °C, but also improved the regrowth rate of in vivo-derived synthetic seeds to 100% when they were stored in 6 °C under light. Exposure to light during storage significantly increased shoot length of regrown synseeds when compared to those stored in darkness. This difference in shoot growth disappeared when synseeds were treated with 25 µM ASA. All regenerated plantlets were rooted and acclimatized in sterile rockwool plugs without morphological changes.

## 1. Introduction

Legalization or decriminalization of cannabis (*Cannabis sativa* L.) has been rapidly increasing worldwide over the past two decades. As of September 2021, over 70 countries have legalized some form of medical use of cannabis, while few countries including Canada, Uruguay, Mexico, The Netherlands, Spain, South Africa, and parts of the United States have legalized cannabis for adult use [1]. The global cannabis market value has been estimated at $214–344 billion USD and legal markets are projected to expand in the next few years [2]. Cannabis predominantly is a dioecious cross-pollinated plant, and its female flower is richest in acidic form cannabinoids including Δ^9^-tetrahydrocannabinolic acid (THCa) and cannabidiolic acid (CBDa) [3]. These compounds and derivates thereof are involved in the treatment of disease conditions such as cancer, Alzheimer’s, multiple sclerosis, chronic pain and inflammation, glaucoma, and many others [4]. A recent study revealed that cannabinoids block cellular entry of multiple SARS-CoV-2 variants and exhibited a potential to prevent as well as treat SARS-CoV-2 infection [5]. Due to the nature of reproduction system in cannabis (dioecy, cross pollination), seed propagation is not commonly practiced as male plants have no commercial use and lack genetic uniformity among seedlings [6]. Therefore, conventional methods of clonal propagation (stem cuttings) of cannabis became the primary method to maintain and propagate elite cultivars for industrial cultivation [7]. Although clonal propagation through stem cuttings is fairly simple [7], it is beset with numerous difficulties: (i) rooting success from stem cuttings depends on environmental factors and the physiological state of the mother plant, (ii) multiple fungal, bacterial and viral diseases can be transmitted through cuttings [8,9], (iii) maintaining mother plants as a propagule source requires significant space and labour to safeguard from disease and accidental loss [6].

Synthetic seed (synseed) technology could be a valuable aid for clonal propagation at a commercial scale as it provides a basis for the production of pest and disease-free certified clean plant material and germplasm conservation [10]. Synseeds are formed by encapsulation of various plant micropropagules such as somatic embryos [11,12], nodal segments [13], axillary shoot buds [14] and apical meristem tips [15] in a covering gel. Hydrogel medium of calcium alginate is the most widely used matrix, with plant-specific modifications. Usually, combinations of 2–3% sodium alginate with 50–100 mM calcium chloride are implemented to produce calcium alginate matrix to encapsulate axillary buds, nodal segments and shoot tips in various plants [16,17,18,19].

The storage of synseeds at low temperature aims to reduce the metabolic rate of encapsulated propagules and prolong storage time [20]. On the other hand, low temperature treatment may induce chilling stress, triggering elevated levels of reactive oxygen species (ROS) and cause injury in propagules during storage, especially in tropical species [21,22]. ROS triggers the biosynthesis of stress hormone salicylic acid, in turn the level of glutathione increases which will aid in ROS scavenging to alleviate biotic and abiotic stresses [23]. It has been shown that commonly elevated ROS levels upon the flooding, salinity, and cold stresses are regulated through salicylic acid (SA)-mediated homeostasis [24].

The study of Cannabis encapsulation is limited to a few papers thus far. Lata et al. [14] utilized non-embryogenic in vitro-derived micropropagules for production of cannabis synthetic seeds. The regrowth and conversion rates right after encapsulation without storage time were assessed under in vitro and in vivo conditions. Later, the same group showed the genetic fidelity of cannabis plants derived from synseeds [25] and 60% success rate when they store synseed for 24 weeks at 15 °C [26].

Present study aims to establish an effective and affordable protocol for commercial cannabis synseed production using in vitro and in vivo-derived nodal segments of elite cultivar ‘Slurricane’. Synseed viability under various treatments and time periods up to 150 days were verified as an alternative solution for large-scale propagation and germplasm conservation.

## 2. Results and Discussion

### 2.1. Explant Source, Encapsulation Matrix, and Impact of Storage Temperature on Synseed Viability

**Figure 1:**
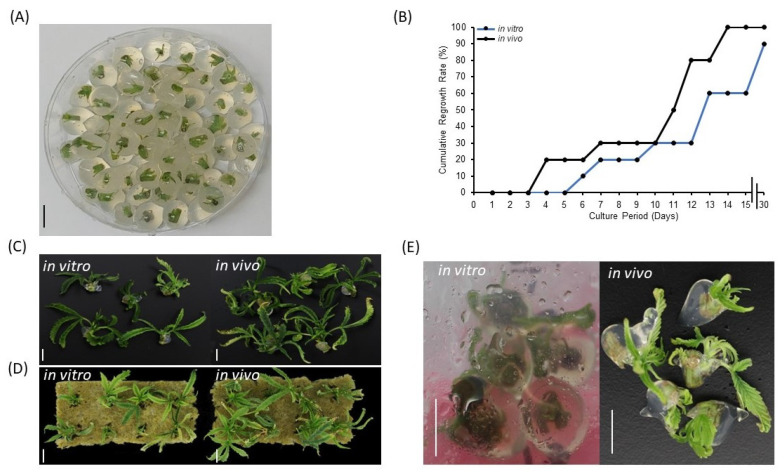
Regrowth of cannabis synseeds. Encapsulated nodal segments using calcium alginate matrix to yield 10 mm cannabis synseeds (**A**), regrowth rates of in vitro- and in vivo-derived synseeds on MS medium without a storage period up to 30 days culture (**B**), plantlets after 30 days of regrowth on MS Cannabis medium (**C**), and on sterile rockwool (**D**). Precocious germination of in vitro and in vivo-derived synseeds during the first 15 days of storage at 22 °C (**E**). Scale bars represent 1 cm.

Encapsulation of synthetic seeds requires a balance of slenderness and rigidity to contain an explant without hindering regrowth. Apart from its physical characteristics, the encapsulation matrix provides moisture and nutrient for axillary bud regrowth. To determine a superior plant material source and expand options for cannabis synseed production, currently growing plants in tissue culture vessels (in vitro) and a grow room (in vivo) were used. In the current study, treating 5 mm nodal segments either from in vitro or in vivo sources with 3% alginic acid sodium salt and 75 mM calcium chloride has provided 10 mm diameter synseeds with the most suitable gelling matrix for the formation of uniform and easy to handle beads (Figure 1A). In vivo-derived synseeds without storage time were germinated to 100% on Murashige and Skoog (MS) medium within 15 days culture, whereas regrowth rate of in vitro-derived synseeds lag behind, reaching only 60% and 90% after 15 and 30 days culture, respectively (Figure 1B). In vivo-derived synseeds without storage time produced larger plantlets than in vitro in MS and rockwool media (Figure 1C,D). This suggests that (i) the selected concentration of alginic acid sodium salt and calcium chloride is suitable for germination of nodal segments of cannabis stem regardless the source of explants. (ii) In vivo-derived explants are thicker segments and enriched with endogenous nutrients and display higher regrowth rate and larger plantlets. The majority of synseed studies are focused on encapsulation of in vitro-derived propagules, however, the possibility of encapsulation of in vivo-derived propagules is limited to few crops [27,28]. Our findings demonstrated that indoor grown cannabis mother plants can be directly used for synseed production with high storage capacity which eliminates dependence on plant tissue stock. Although, in vivo derived explants do not require a 4 wk pre-culture period in vitro, surface sterilization should be applied prior to encapsulation. Furthermore, we observed that synseeds from in vitro and in vivo sources displayed precocious germination when stored at 22 °C for 15 days (Figure 1E). Precocious germination could be deteriorative for synseed medium-term storage. Environmental conditions that slow down the cellular metabolism or put synseeds in a quiescent stage are vital for preserving regrowth ability after extended storage periods [29]. In this regard, temperature plays a crucial role in maintaining dormancy of the explant. We found that precocious germination of synseed was delayed when they were stored at 6 °C (Supplementary Materials, Figure S1). Therefore, subsequent storage experiments were all conducted at 6 °C storage temperature to maintain buds of nodal segments in the dormant status. Similar findings on precocious germination of synseed was observed in neem (*Azadirachta indica*) at 23 °C [30]. However, no regrowth was observed when the synseeds were incubated at 4 or 8 °C for 1 month [30]. Low temperature (5 °C) was used on cannabis synseeds during 6 months storage with relatively low survival rate (43%) [26]. This rate improved to 60% when synseed stored at 15 °C [26]. This seems to be a feature of tropical and subtropical plants as chilling injury is significant below 12 °C [31]. Therefore, mitigation of chilling injury is required to expand the viability of synseed stored at low temperature.

### 2.2. Impact of Light on Cannabis Synseeds Viability and Regrowth Rates

**Figure 2:**
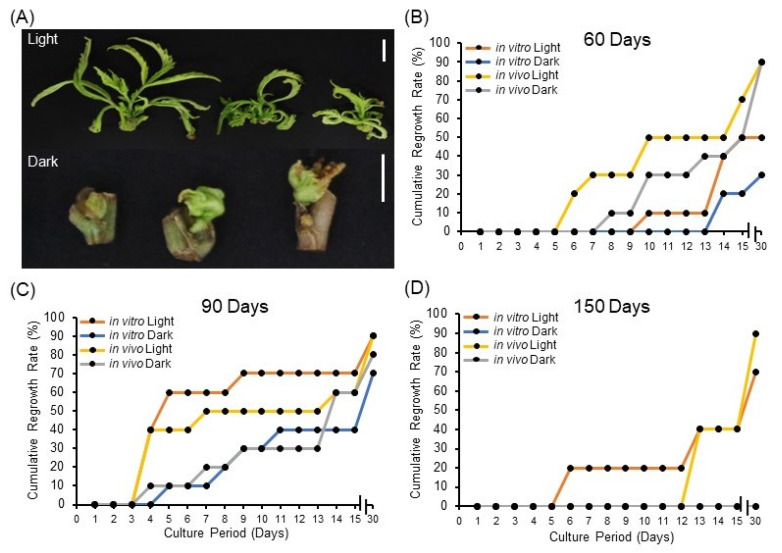
Impact of light exposure on cannabis synseed regrowth rate. Representative regrowth of plantlets after 60 days of light or dark storage at 6 °C followed by 15 days culture (**A**). Thirty-day cumulative regrowth rates of in vitro and in vivo-derived synseeds on MS culture medium after 60 (**B**), 90 (**C**), and 150 days (**D**) storage time at 6 °C under 50 μmol s^−1^ m^−2^ light and an 18/6 h photoperiod or dark conditions. Scale bars represent 1 cm.

Here, we investigated the effect of light exposure during storage on synseed regrowth rate. It appears that synseeds exposed to 50 μmol s^−1^ m^−2^ light have advanced regrowth rate of axillary buds after 60 and 90 days of storage (Figure 2A–C) regardless of explant source. Interestingly, in vitro and in vivo-derived synseeds stored in the dark for 150 days did not germinate, whereas synseeds exposed to light for 150 days yielded 75% and 90% regrowth, respectively (Figure 2D). This suggests the positive impact of light on synseed viability during the storage period, hence light exposure should be considered as a critical parameter in setting up synseed storage facilities. Furthermore, 60- and 90-day stored synseeds displayed reduced growth rate differences among treatments after 30 days culture compared to 15 days (Figure 2B,C). It is tempting to mention that light promotes bud development and photosynthesis, making synseeds ready to germinate once they are placed in standard culture conditions. This role was even more crucial for medium-term storage (150 days, Figure 2D) when endogenous nutrients were depleted. The impact of light on synseed viability and bud development seems to be species specific. *Taraxacum pieninicum* synseeds stored at 4 °C in the dark had higher viability than those exposed to light after 6 and 9 months of storage [32]. This study showed that the negative effect of light due to elevated abscisic acid (ABA) upon medium-term storage [32,33]. On the other hand, *Hibiscus moscheutos* encapsulated nodal segments stored at 5 °C for 6 months showed no regrowth differences between light and dark storage whereas light exposure during 25 °C storage led to advance regrowth rates [34]. This finding is aligned with commercial cannabis propagation practices that involve light exposure to stem cuttings for extended hours (18 h) for the first 21 days to induce breakage of axillary bud dormancy and initiate sprouting [35]. Our findings showed that light play a pivotal role on viability of cannabis synseeds during medium-term storage.

### 2.3. Acetylsalicylic Acid Conditionally Alters Regrowth Rate of Cannabis Synseed

Next, to mitigate chilling injury of cannabis synseeds during storage and to scavenge ROS from the encapsulated explant, we investigated the impact of acetylsalicylic acid (ASA) in the encapsulation matrix aiming to preserve the viability and regrowth rate of synseeds. We hypothesized that adding ASA in the encapsulation matrix extends the viability of synseeds at low temperatures. The addition of ASA in combination with other storage conditions, such as low temperature and light, is investigated in this section.

**Figure 3:**
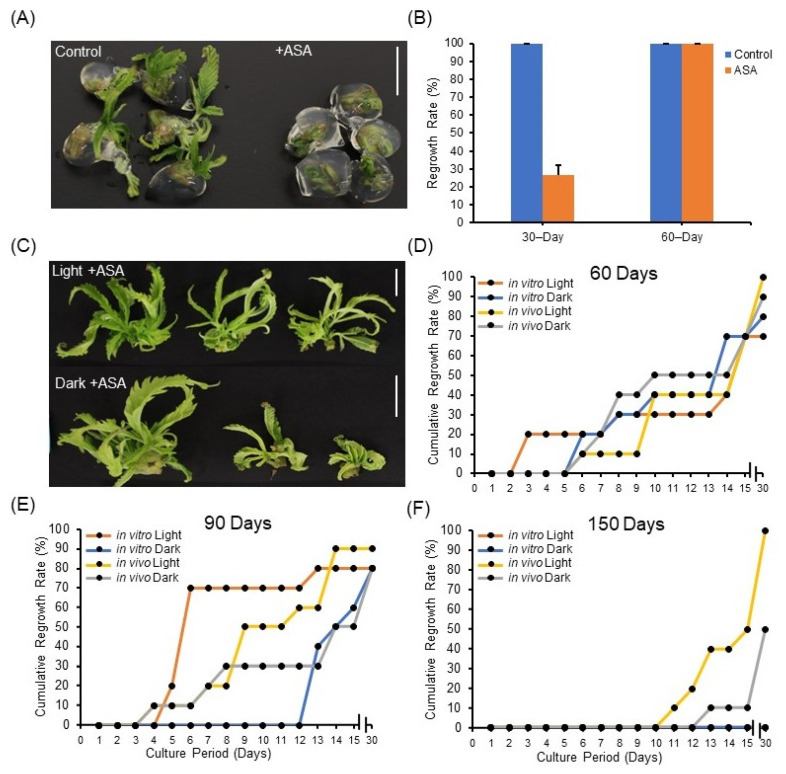
Impact of acetylsalicylic acid (ASA) on synseed storage and regrowth rates. Representative synseeds (**A**) and the corresponding precocious germination rate during 30 and 60 days of storage at 22 °C with or without 25 µM ASA (**B**). Representative synseeds supplemented with 25 µM ASA and stored for 60 days light or dark and regrown for 30 days on MS medium (**C**). Thirty-day cumulative regrowth rates of 25 µM ASA treated synseeds derived from in vitro or in vivo plants after 60 (**D**), 90 (**E**), and 150 day (**F**) storage at 6 °C under 50 μmol s^−1^ m^−2^ light and an 18/6 h photoperiod or dark exposure. Scale bars represent 1 cm.

First, ASA was supplied directly in the encapsulation matrix of synseeds stored at 22 °C. Our results show that precocious regrowth under 22 °C storage was retarded when the encapsulation matrix was supplied with ASA (Figure 3A,B), suggesting low temperature (6 °C) can be substituted by addition of ASA into the encapsulation matrix for short-term storage. This could benefit synseed import and export where controlling temperature during transportation is difficult.

Additionally, explant source and light exposure had minimal impact on regrowth rates of ASA treated synseeds stored for 60 and 90 days (Figure 3C–E). ASA treatment of 60-day stored in vitro synseeds corresponded to a pronounced regrowth rate improvement over control from 30% to 80% and 50% to 70%, when stored in the dark or light, respectively (Figure 2B and Figure 3D). The difference in regrowth rates between ASA treated and untreated was negligible in synseeds stored for 90 days (Figure 2C and Figure 3E). ASA treatment during 150 days storage increased the regrowth rate of in vivo-derived dark stored synseeds from 0% to 50% whereas in vitro-derived light stored synseeds did not germinate (Figure 2D and Figure 3F). ASA treatment of in vivo-derived, light stored cannabis synseeds maintained viability and regrowth up to 100% after 150 days (Figure 3F).

A previous study showed that bean and tomato seeds imbibed with ASA solution prior to sowing enhanced tolerance to chilling [36]. This suggests the improved germination of ASA treated synseeds at 6 °C could be associated with the reduction in chilling injury. Sunflower (*Helianthus annuus* L.) and *Casimairoa edulis* shoot tips were encapsulated and stored with 50 µM salicylic acid for 90 days in the dark at 4 °C and viability percent increased from 33 to 59% and 40% to 80%, respectively, compared to controls [37,38]. Similarly, encapsulated neem tree nodal cuttings had an increase in germination rate from 19% to 75% when treated with ASA prior to encapsulation and stored at 12 °C, while a lower (4 °C and 8 °C) temperature treatment significantly hindered germination [30]. These findings align with our observations which states that synseeds treated with ASA in the dark have improved longevity and higher regrowth rate compared to control (Figure 3F).

### 2.4. ASA Improves Regrowth Vigor of Dark Stored Synseeds

**Figure 4:**
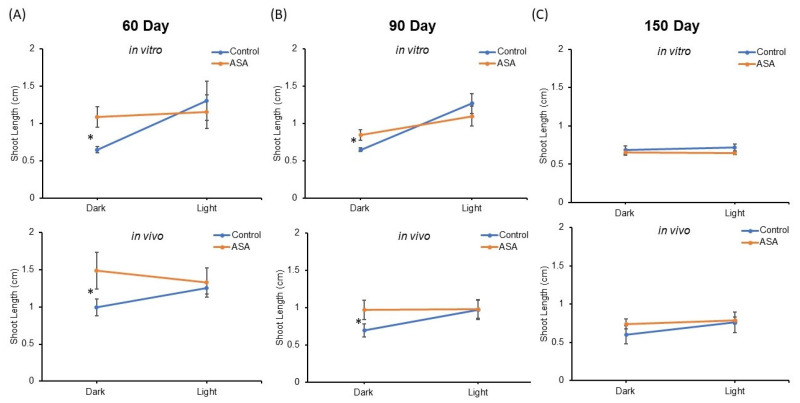
Comparison of shoot length after 15 days culture on MS medium. Synseeds derived from in vitro or in vivo plants were stored at 6 °C for 60 (**A**), 90 (**B**), or 150 (**C**) days under 50 μmol s^−1^ m^−2^ light and an 18/6 h photoperiod or dark conditions with or without 25 µM acetylsalicylic acid (ASA). Error bars represent standard error (*n* = 5 ≤ 10). Two factor ANOVA model was used to identify an interaction between light and ASA treatments. Differences between mean shoot length are compared with Tukey’s test denoted by an asterisk (*p*-value ≤ 0.05).

Shoot length means of encapsulated nodal segments after 15 days regrowth were compared in all treatments (Figure 4). Mean shoot length between synseeds derived from in vitro and in vivo sources were not significantly different and no significant interactor with source of synseeds was observed by ANOVA analysis. Light exposure and ASA supplementation for 60 and 90 days of storage display a significant interaction effect on shoot length after the regrowth period (*p*-value ≤ 0.05, Figure 4A,B).

ASA supplementation in the encapsulation matrix provided significantly higher shoot lengths than untreated synseeds when stored in the dark, whereas no significant difference was found between treated and untreated under light exposure. This suggests light treatment during storage can be substituted by ASA supplementation at least for 60 and 90 days storage. The presence of ASA in the encapsulation matrix offers better protection during cold storage as seen by significantly improved regrowth rates and shoot lengths (Figure 3 and Figure 4).

### 2.5. Rooting and Establishment of Synseed Shoots

**Figure 5:**
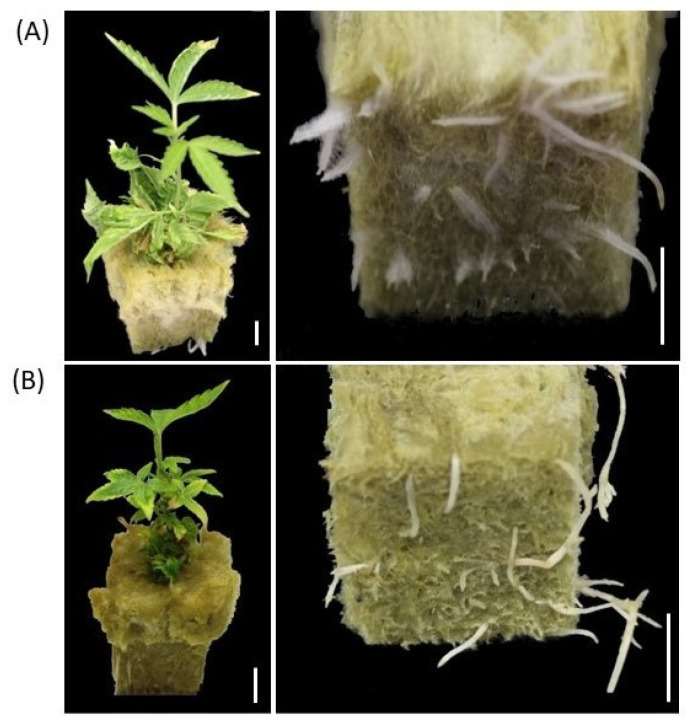
Germination of synseeds. Rooted plants originated either from synseeds that were stored for 90 days under 50 μmol s^−1^ m^−2^ light, 18/6 h photoperiod at 6 °C and regrown on MS medium for 15 days (**A**) or micropropagation methods as described by Zarei et al. 2022 (**B**) followed by 40 days rooting period in rockwool plugs. Scale bars represent 1 cm.

Shoots of germinated synseeds on MS culture media were dissected to 2.5–3 cm and placed in sterile rockwool to root using photoautotrophic micropropagation as described elsewhere [39,40]. The regenerated plantlets were rooted after 4–6 weeks in rockwool plugs and acclimated by transferring the rooted plants to a standard tray under a transparent dome within a week (Figure 5A). Acclimated cannabis plants originated from synseeds did not display any form of leaf malformation and were indistinguishable from conventionally propagated plantlets in their visual phenotype (Figure 5).

## 3. Materials and Methods

### 3.1. Plant Material and Environmental Conditions

In vitro and in vivo-derived plant materials were obtained from the Safari Flower Co. micropropagation lab and indoor mother room, respectively. An elite cannabis cultivar ‘Slurricane’ was utilized in this study. Stock shoot cultures of in vitro cannabis were grown for 4 wk in Magenta GA7 boxes (Magenta Corp., Chicago, IL, USA) containing 40 mL MS medium with vitamins, 2 µM mT (PhytoTechnology Lab, Shawnee, Mission, KS, USA), 1% agar, 3% sucrose and 1 mM 2-(N-morpholino) ethanesulfonic acid (MES) at pH 5.6. The plantlets in micropropagation lab were maintained at 22 ± 2 °C, 45–55% relative humidity (RH), 18/6 h light/dark photoperiod, photosynthetic photon flux density (PPDF) of 50 μmol s^−1^ m^−2^, and 900–1100 µmol mol^−1^ CO_2_. The indoor mother room was maintained at 23 ± 3 °C, 55–65% RH, 800 μmol s^−1^ m^−2^ PPFD and 900–1100 µmol mol^−1^ CO_2_. Dimmable light emitting diode tubes (Infinity 2.0, Thrive Agritch, New York, NY, USA) were used in the micropropagation lab and 1000 W Fusion Bright high-pressure sodium light (Grow light, Beamsville, ON, Canada) were used in the indoor mother room.

### 3.2. Explant Preparations

In vivo-derived 10 cm shoot cuttings were surface sterilized, as described by Zarei et al. [39]. In brief, the leaves on cuttings were trimmed off, and the stems were first rinsed under running tap water for 10 min, immersed in 60% ethanol for 30 s, followed by treatment with a 10% household bleach (0.62% active chlorine) and 0.1% Tween 20 (Fisher Scientific, Mississauga, ON, Canada) solution. The closed vessel containing cuttings and the sterilization solution were placed on a rotary shaker (Gyromax 838, Amerex Instruments, Inc. Concord, CA, USA) at 60 RPM for 22 min. Cuttings were thoroughly rinsed three times with sterile reverse osmosis (RO) water under laminar flow.

### 3.3. Encapsulation Matrix

Nodal segments (5 mm) containing single axillary buds were fully submerged in 3% alginic acid sodium salt (Acros Organics, Geel, Belgium) with full-strength Murashige and Skoog (MS) basal salt medium (PhytoTech Labs, Lenexa, KS, USA). Alginic acid coated buds were submerged into 75 mM calcium chloride (PhytoTech Labs) with MS medium for 30 min incubation to harden followed by three times rinsing in sterile RO water. To examine the impact of ASA (PhytoTech Labs) on synseed quality and regrowth rate, 25 µM ASA was added to the MS basal salt medium that carries alginic acid and calcium chloride.

### 3.4. Storage of Encapsulated Seeds and Treatments

Synseeds were kept in 10 mL test tubes, capped, and sealed with parafilm. Four tubes with five synseeds per tube were placed in 335 mL volume magenta GA-7 plant tissue culture vessels (PhytoTech Labs) with a water-moistened paper towel to maintain high RH. Light and dark treatments were applied by exposing synseeds to 50 μmol s^−1^ m^−2^ PPFD with 18/6 h light/dark photoperiod or covering vessels with aluminum foil, respectively. Two temperature treatments, 6 ± 1 °C or 22 ± 2 °C, were adjusted by a laboratory fridge (FCGM181RQB, Frigidaire, Mississauga, ON, Canada) or an automated indoor climate (Hoogendoorn, America Inc., Vineland Station, ON, Canada), respectively.

### 3.5. Regrowth of Cannabis Encapsulated Seeds and Data Collection and Analysis

Cannabis synseeds were set to regrow either in a semi-solid MS cannabis multiplication basal salt mixture with 2 µM *meta*-Topolin (PhytoTech Labs) or in sterile rockwool plugs (AO′K, 1.5″ × 1.5″ square × 1.57″H, Grodan Inc., Milton, ON, Canada) which were placed in OV80 culture vessels (OV80, Sac O_2_^©^, Deinze, Belgium). The media was set to pH 5.6 before autoclaving and supplemented with 1% agar and 3% sucrose. Fertilizer solution was added into rockwool plugs to half of its saturation as described previously [39,40]. Regrowth of synseeds was recorded daily for up to 15 and 30 days. It was defined as buds protruding the encapsulation matrix and develop at least one fully unfurled leaf with more than one leaflet. The experiment was conducted as a randomized block design. The cumulative regrowth rates were determined from 10 pooled synseeds collected from 2 culture vessels. Shoot length was obtained by taking images of synseeds after 15 days of regrowth using ImageJ software (NIH, Bethesda, MD, USA). Two-way analysis of variance (ANOVA) was performed to compare the interaction effect of light exposure and ASA supplement on shoot length. Tukey’s test was applied to identify significant *P*-values between multiple means. RStudio (Build 576, R 4.2.1) was used to compute ANOVA models and Tukey’s test [41]. Cumulative regrowth rate and shoot length interaction plots were visualized using Microsoft Excel (Version 2209).

## 4. Conclusions

The results of this study introduce a method using nodal segments of female cannabis with 100% regrowth rate after 150 days of storage under in vitro conditions. Ultimately, the impact of balanced matrix composition, explant source, temperature, light exposure, and media supplementation during storage were investigated to expand viability of cannabis synseeds during storage. Hence, this feasible technology can be considered as a promising strategy not only to conserve and distribute cannabis germplasm, minimizing pests and pathogen distribution, but also to mass propagate cannabis cultivars at the commercial scale without being dependent on tissue culture plantlets.

## Data Availability

Research data are available from the authors.

## Supplementary Materials

The following supporting information can be downloaded at: https://www.mdpi.com/article/10.3390/plants11233186/s1, Figure S1: Synseeds with or without 25 µM ASA after 30 days of storage at 6 °C.
