# Supplementary material for: Cannabis Synthetic Seeds: An Alternative Approach for Commercial Scale of Clonal Propagation and Germplasm Conservation
Source: Plants (Basel). 2022 Nov 22;11(23):3186. doi: 10.3390/plants11233186 (PMC9738115; doi:10.3390/plants11233186)
Supplement: Supplementary file 1 [file plants-11-03186-s001.zip › plants-2041443-supplementary.pdf]

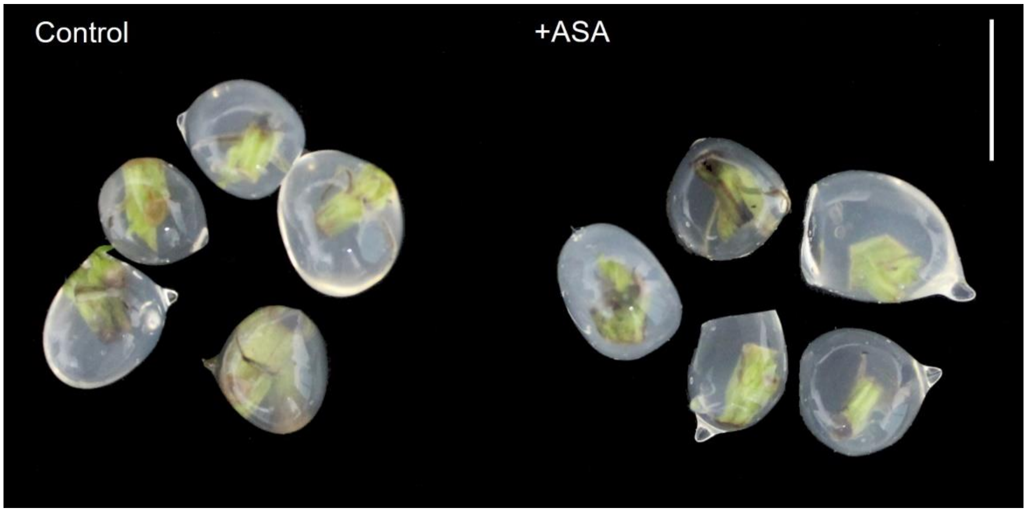

Supplementary Figure S1. Synseeds with or without 25 μM ASA after 30 days of storage at 6 °C. Scale bars represent 1 cm.
